# Laboratory Methods in Relation to Tuberculosis

**Published:** 1913-04-12

**Authors:** 


### THE CLINICAL LABORATORY.

### V. Laboratory Methods in Relation to Tuberculosis.

### II.—SPUTUM.

The simple procedure of staining a film of sputum by the Ziehl-Neelsen method is one of the most valuable diagnostic measures of modern medicine. There is nothing to add to the descriptions of technique as given in almost every text-book. The three points of greatest importance are to select a suitable particle of sputum, to over-stain, in the first instance, with carbol-fuchsin, and then to make a very careful search for bacilli in the stained film.

Opinions differ as to the value to be attached to sputum examination. Many refuse to make a positive diagnosis of pulmonary tuberculosis until bacilli have been found in the sputum, whilst others endeavour to diagnose the disease before the bacilli have appeared in the sputum, and whilst the disease is still in the "closed" stage. In cases of emphysema, bronchitis, pneumokoniosis, and bronchiectasis, complicated by tuberculosis, it is almost impossible to make a positive diagnosis of tuberculosis without the presence of tubercle bacilli in the sputum. This is granted by most clinicians, but the crucial question is: Can a confident diagnosis of pulmonary tuberculosis be made in the case of a young person with otherwise healthy lungs before bacilli are found in the sputum? It seems that in a very small percentage of cases this can be done, but in the majority of cases the detection of the bacilli is the first positive sign of tuberculosis. It is well known that a large number of "cured" cases of consumption have never had bacilli in the sputum, and it appears probable that many of these cases have never required treatment. In the German Army, where every endeavour is made to make the diagnosis at the earliest possible moment, it is stated that 93 per cent. of all cases have bacilli in the sputum. In some of the sanatoria of Germany the proportion of cases with bacilli is very small, and one hears that the Army authorities have invaded the sanatoria and taken the patients to serve in the Army. This action should result in the collection of very valuable data, for the diagnosis of tuberculosis apart from bacteriological examination is full of pitfalls.

The greatest difficulty encountered in bacteriological diagnosis is the fact that bacilli may be so few that they are overlooked, and methods have been devised for concentrating into one microscopical preparation the bacilli from a large quantity of sputum. Cultural media have been evolved which, by encouraging the growth of the tubercle bacillus and inhibiting the growth of others, serve the same purpose as does Löffler's blood serum in diphtheria diagnosis. They have never come into extensive use, and of late years have been superseded by chemical methods, whereby the sputum is rendered fluid, so that the bacilli may be separated by centrifugalisation. The *antiformin* method is the one in most general use. Antiformin is a proprietary preparation which may be replaced by a mixture of equal parts of the liquor sodæ chlorinatæ (B.P.) and a 15 per cent. solution of caustic soda. There are some minor differences of technique, but the following will be found a rapid and reliable method, which at the same time avoids the necessity of working with living bacilli. The whole or part of the twenty-four hours' sputum is shaken up with an equal volume of 15 per cent. antiformin solution in fresh distilled water. When homogeneous this is boiled and then centrifugalised. There results a small deposit which contains the bacilli. This may be stained, but as the material is very apt to come off the slide during treatment with the acid, it is advisable to wash with some distilled water or saline made from distilled water. The results are a little superior to the simple staining of a film. Macalister, at the Lister Institute, in a very long series of cases, found an improvement of only 0.54 per cent. Other workers have produced much better results; but it is quite possible that they are due to the fact that the ordinary technique was not so good as the routine technique at the Lister Institute. It has also been shown that acid-fast bacilli may be found in tap water, and therefore, unless distilled water be used for dilution and washing, the antiformin method is unreliable. Some recent work on the use of antiformin for blood examination demonstrates a serious possibility of error. By means of antiformin "tubercle bacilli" were found in the blood of a large proportion of early cases of phthisis, whilst the results of animal inoculation were negative. Subsequent work has shown that *normal* blood, after treatment with antiformin, shows the presence of "tubercle bacilli." From this it appears that antiformin may produce "artefacts" from blood which have the appearance of tubercle bacilli. In place of the above technique one may incubate sputum for two or three hours with one-fourth its volume of antiformin. The bacilli are separated by the centrifuge and washed several times in sterile water, and then cultivated on special media or, better, inoculated into the guinea-pig. This method appears to be the ideal one, but has not yet been used to any great extent.

When it is suspected that a child or an adult is swallowing sputum, none of which can be obtained for examination, the fæces should be examined by the antiformin method. Two successive treatments are required before a deposit suitable for staining can be obtained.

### Urine.

The diagnosis of renal tuberculosis is always difficult, and the clinical diagnosis will always be dependent on bacteriology. Although the discovery of tubercle bacilli in the urine is not absolutely diagnostic of tuberculous lesions within the urinary tract, bacilli being sometimes found in the urine of patients suffering from some tuberculous lesions elsewhere in the body, yet the association of symptoms of renal tuberculosis with the passage of bacilli in the urine is more than sufficient for a diagnosis.

There is one great source of error, and only one, but it has been responsible for many grave errors of diagnosis. The mucous membranes of the external genitals, in both the male and female, contain large numbers of acid-fast bacilli (*Smegma bacillus*) which in their morphological and staining properties are very similar to the tubercle bacillus. It is true that in many cases the two organisms can be distinguished by appropriate staining methods in the hands of an expert, but this more complicated technique is far from infallible, and there are instances where the best have made mistakes. If proper care be applied to the collection of the specimen they are not necessary, and it is a maxim of bacteriology that it is always sounder practice to avoid contamination than to attempt to distinguish contaminating organisms from pathogenic ones. In the male the external genitals should be carefully washed, and then the specimen carefully passed into two vessels; the contents of the second vessel (which should have been previously sterilised) are alone used for the examination. In the case of the female a catheter specimen is absolutely necessary, and extreme care must be taken that it is not accidentally contaminated by vulval secretion. Two nurses are required. One of them washes the external genitals, and then holds apart the labia, so that the second nurse holding a sterile catheter (armed with a continuation tube passing into a sterile vessel) in her gloved hand can pass the point of the instrument directly into the urethra without there being any contact with the vulva. These precautions may seem extreme, but experience has shown that they are all necessary. The examination of the specimen is a fairly simple matter. The deposit from a large quantity of urine is collected by means of a good electrical or hydraulic centrifuge, spread on films and stained in the ordinary manner. There is one further precaution. If the microscope reveals the presence of any large vaginal epithelial cells one should refuse to accept the specimen as uncontaminated. It is the personal opinion of the present writer that an animal experiment should be used in all doubtful cases.

### Cerebro-Spinal Fluid.

The diagnosis of tuberculous meningitis can often be clinched by the discovery of the tubercle bacillus in the cerebro-spinal fluid. The deposit obtained by the centrifuge should be stained by the ordinary method. In some cases the bacilli cannot be demonstrated, and in these the only "laboratory" evidence is the presence of a lymphocytosis in the fluid. Tubercule bacilli have been found in the cerebro-spinal fluid, where a subsequent *post-mortem* examination has revealed a complete freedom from tuberculous meningitis, although there have been other tuberculous lesions in the body. This suggests that in the case of advanced tuberculous lesion there may be a general flooding of the *tissues* and blood with tubercle bacilli, for it is now well recognised that in the terminal stage of pulmonary tuberculosis there is often a bacteræmia. Another interesting fact is that there have been recorded a few cases where recovery from tuberculous meningitis has ensued, even when the diagnosis has been confirmed by the discovery of the bacillus in the cerebro-spinal fluid.

